# Prevalence of Active Syphilis Infection and Risk Factors among HIV-Positive MSM in Zhejiang, China in 2015: A Cross-Sectional Study

**DOI:** 10.3390/ijerph16091507

**Published:** 2019-04-28

**Authors:** Lin Chen, Jiezhe Yang, Qiaoqin Ma, Xiaohong Pan

**Affiliations:** Zhejiang Provincial Center for Disease Control and Prevention, 3399 Binsheng Road, Hangzhou 310051, Zhejiang, China; lchen@cdc.zj.cn (L.C.); jzhyang@cdc.zj.cn (J.Y.); qqma@cdc.zj.cn (Q.M.)

**Keywords:** HIV, acquired immunodeficiency syndrome, syphilis, cross-sectional studies

## Abstract

Objectives: To determine the prevalence of active syphilis infection and explore the risk factors for active syphilis in human immunodeficiency virus (HIV)-positive men who had sex with men (MSM) in Zhejiang Province, 2015. Design: Data on HIV-positive MSM living in Zhejiang Province were obtained from the National Center for AIDS/STD Control and Prevention (NCAIDS) reporting system and the Zhejiang provincial AIDS/STD surveillance system between June and December 2015. The information included risky behavior, years with diagnosed HIV, and highly active antiretroviral therapy (HAART). SPSS 19.0 was used for the data analysis. Results: The analysis included 3616 MSM. Of these, 11.3% (407/3616) had active syphilis. Multivariate logistic regression analysis revealed that HAART was significantly associated with an increased risk of active syphilis infection (odds ratio (OR) = 1.760, 95% confidence interval (CI) 1.187–2.611). Compared with participants diagnosed with HIV for <2 years, patients diagnosed with HIV for >5 years had a higher risk of active syphilis infection (OR = 1.707, 95% CI 1.167–2.495). Age and number of sex partners were also independent risk factors for active syphilis infection. Conclusions: The incidence of active syphilis infection is high among HIV-positive MSM in Zhejiang Province; age, number of sex partners, years with diagnosed HIV, and receiving HAART were risk factors. Patients who are elderly, have lived with HIV for a longer period, have more sex partners, and receive HAART should be the focus of interventions to promote changes in behavior and decrease syphilis infection.

## 1. Introduction

Human immunodeficiency virus (HIV) prevalence has been rapidly increasing in men who have sex with men (MSM) in the last decade, despite an increase in the use of highly active antiretroviral therapy (HAART) [1,2,3]. According to estimates of the Chinese Center for Disease Control and Prevention (China CDC), there were more than 780,000 people living with HIV (PLWH) in 2011 and 17.6% became infected through male-to-male sexual contact behavior [4]. Furthermore, one of the challenges among MSM is the high prevalence of co-infection with HIV and sexually transmitted infections (STIs), especially syphilis [5,6,7,8]. The relationship between HIV and STIs has been revealed in many studies [9,10].

Individual and public health benefits can be achieved through syphilis screening and testing [11,12]. It has been well established that syphilis screening reduces the possibility of HIV infection. Screening for syphilis infection also revealed that common risky behaviors, such as sex without a condom, still exists among MSM living with HIV. However, because of reporting bias this key information could not be collected. Although, implementing syphilis testing helped to partially solve this issue.

Zhejiang Province is a modern area with many migrants moving in and out. The sixth census in 2010 reported 56 million permanent residents and 15 million migrants [13]. At the end of 2015, a total of 15,299 PLWH were living in Zhejiang Province and 6323 were MSM. Male-to-male sexual contact has become the main route of HIV transmission in Zhejiang [2]. Among newly diagnosed HIV patients, the proportion of patients in Zhejiang Province infected though male-to-male sexual contact in the last 5 years was 37.8, 41.2, and 40.8% in 2014, 2015, and 2016, respectively (unreported). To respond to this increase, compulsory syphilis screening every 6 months was implemented for HIV-positive MSM in Zhejiang Province in 2011. Hospitals and the China CDC managed the additional follow-up with the HIV patients that was required to implement the new strategy.

Few studies have been conducted to determine the prevalence and risk factors of syphilis infection among HIV-positive MSM in China. The aims of this study were (1) to describe the prevalence of syphilis infection in HIV-positive MSM, (2) to determine risk behaviors in study participants, and (3) to identify independent risk factors of syphilis infection to recommend future interventions.

## 2. Methods and Analysis

### 2.1. Study Design and Data Collection

This was a cross-sectional study conducted in Zhejiang Province between July and December 2015. Criteria for participant enrolment included: (1) an HIV-positive diagnosis before 2015, (2) infection through male-to-male sexual contact, and (3) age >18 years.

Participants’ basic information and data regarding HIV infection, such as risky behavior, year of diagnosis, HAART, and CD4 T cell counts were collected from the National Center for AIDS/STD Control and Prevention (NCAIDS) reporting system. The data in this system are required to be collected during routine tracking and clinical treatment of patients. The forms used for data collection are standardized nationwide and are completed by health care facilities (i.e., hospitals and the local Centers for Disease Control and Prevention (CDC)) every 3 months for AIDS patients and every 6 months for HIV+ patients. The data were downloaded on January 31, 2016. Patients who were not from mainland China or could not be located by the health care facility after diagnosis were excluded. Data on active syphilis infection, which is also reported by health care facilities every 6 months, were collected from the Zhejiang provincial AIDS/STD surveillance system.

Among the patients enrolled in this study, 83.8% (3616/4315) had information on syphilis testing and were included in the analysis.

### 2.2. Ethical Considerations

This study was reviewed and approved by the Institutional Review Board of the Zhejiang Provincial Center for Disease Control and Prevention (Zhejiang CDC; IRB approval number: 2013-001).

### 2.3. Active Syphilis Infection

Syphilis infection was diagnosed through rapid plasma regain (RPR) and the Treponema pallidum particle agglutination test (TPPA). Syphilis infection was considered active if both the RPR and TPPA were positive, which indicated a new infection.

### 2.4. Statistical Analysis

The data were analyzed using SPSS statistical software (version 19.0, IBM, Armonk, NY, USA) Descriptive analyses were used to describe the demographic characteristics and prevalence of syphilis infection among the study participants. The χ^2^ test was used to examine the relationships between demographic characteristics, risky behavior, HIV infection, and syphilis infection. We also performed backward multivariate logistic regression analyses to examine the associations between active syphilis infection and various parameters. The model included all variables: age, marital status, education, mobility, area of registry, condom use with every intercourse in the past 6 months, number of sex partners in the last 3 months, disease progression, years with diagnosed HIV, CD4 T cell count in the last 3 months, and HAART. We reported the 95% CIs and p-values. p-values < 0.05 were considered statistically significant.

## 3. Results

### 3.1. Sample Characteristics

The 3616 individuals enrolled in the study had a mean age of 35.5 (range 18–81) years. In total, 626 (17.3%) were more than 45 years old, 830 (23%) were married, and 2193 (60.6%) had an education level of senior high school or higher. Most were registered in Zhejiang Province (2289, 63.3%), and 36.7% (1327) of the HIV patients migrated between provinces, 11.2% (406) migrated within the province, and 52.1% (1883) of the patients were residing locally. Please refer to Table 1 for additional details.

Of the 3616 MSM, 1383 (38.2%) were diagnosed with acquired immune deficiency syndrome (AIDS). The years with diagnosed HIV ranged from 1 to 13 years, with a mean of 3 years. In total, 28.6% (1035) of the participants had a CD4+ T cell count >500/mm^3^.

In total, 3616 HIV-positive MSM study participants had completed syphilis tests. Among them, 407 (11.3%) were diagnosed with active syphilis infection.

Among all cases, HIV-positive MSM had increased active syphilis infection with increasing age (χ^2^ = 18.214, p < 0.001) (Table 1). The proportion of active syphilis infection among study participants with 0–5 sex partners in the prior 3 months was lower (9.3%) compared with those with 6–10 sex partners (13%) and >10 sex partners (17.2%) (χ^2^ = 28.459, p < 0.001, Table 1). The rate of active syphilis infection was higher in study participants with AIDS compared to those with HIV (12.7% vs. 10.4%, χ^2^ = 4.383, p = 0.036). Study participants with more years with diagnosed HIV had higher rates of active syphilis infection (χ^2^ = 14.259, p < 0.001). Participants taking HAART had higher active syphilis infection rates than those not receiving HAART (χ^2^ = 12.349, p < 0.001).

### 3.2. Multivariate Logistic Regression Analyses of Risk Factors Associated with Active Syphilis Infection

In the multivariate logistic regression analyses, the results showed that older age, years with diagnosed HIV, having more sex partners, and receiving HAART were independent risk factors for active syphilis infection. The MSM patients aged 26–35 years (Ajusted odds ratio (AOR) = 1.948, 95% Confidence interval (CI) 1.224–3.100), 36–45 years (AOR = 1.815, 95% CI 1.109–2.970), and >45 years (AOR = 1.884, 95% CI 1.120–3.168) were more likely to have active syphilis infection compared with participants aged 18–25 years old. Compared with participants who had been diagnosed with HIV infection for <2 years, the risk of active syphilis infection was 1.707 times higher in participants diagnosed with HIV infection for >5 years (95% CI 1.167–2.495). Compared with participants who reported ≤5 sex partners, those who reported 6–10 sex partners (AOR = 1.658, 95% CI 1.243–2.212) or >10 sex partners (AOR = 2.455, 95% CI 1.805–3.341) in the last 3 months were more likely to be diagnosed with active syphilis infection. HAART was also significantly associated with an increased risk of active syphilis infection (AOR = 1.581, 95% CI 1.025–2.441). Table 2 shows the adjusted odds rations for the confounding factors.

Among the participants who had been diagnosed with HIV for <2, 2–5, and >5 years, 82.6, 87.5, and 91.7%, respectively, reported receiving HAART (χ^2^ = 29.355, p < 0.001). The proportions of participants receiving HAART who were 18–25, 26–35, 36–45, and >45 years of age were 76.0, 84.3, 89.3, and 92.5%, respectively.

## 4. Discussion

Syphilis infection, especially active syphilis infection, among HIV-positive MSM is often associated with risky sexual behavior and contributes to HIV transmission in the community. Therefore, we conducted this study to explore the risk factors that could be associated with syphilis infection in HIV-positive MSM in Zhejiang Province. This study contributes to the limited number of studies available on large samples in China. In our study, the prevalence of active syphilis infection was 11.3%. In medical literature, there are few studies in China that report syphilis infection in HIV-positive MSM. A study conducted on 264 HIV-positive MSM in Jiangsu Province reported 13.64% with active syphilis infection during follow-up [14]. Another study conducted in Beijing among 2633 MSM newly diagnosed as HIV-positive reported 13.22% with active syphilis infection [15], which is slightly higher than the rate reported in this study.

The prevalence of active syphilis infection is higher in HIV-positive MSM than in the general MSM population, and it ranged from 1.8 to 9.7% over the past 5 years [16,17,18,19]. In Zhejiang Province, the prevalence of active syphilis infection among MSM regardless of HIV status from 2013 to 2015 was 7.17, 7.46, and 5.99%, based on the sentinel surveillance data (unreported). This may be because HIV-positive MSM are more likely to be sexually active and less self-protective than MSM in general.

Our research determined that taking HAART, years diagnosed with HIV, age, and number of sex partners are independent risk factors for active syphilis infection. An important finding is that HAART was significantly associated with an increased risk of active syphilis infection among HIV-positive MSM. Participants may perceive engaging in unprotected sexual behavior as low risk because they are already infected and feeling better. Those receiving HAART may also believe that because they are receiving HAART they are unable to transmit the HIV virus, which is true if they are adhering to their medication regimen. This has been used to motivate PLWH to adhere to their medication regimen, however, it may also contribute to unprotected sex and the increase in STDs. Furthermore, receiving HAART decreases the mortality rate and improves quality of life, which may lead to higher rates of sexual activity [20,21]. In China, the enrolment criteria for HAART changed from a CD4 count of ≤200/mm^3^ before 2011 to a CD4 count <350/mm^3^ for 2011–2014. As of 2015, patients can elect to receive HAART once diagnosed as HIV-positive. The epidemic of HIV and STIs will face new challenges with this new policy. New strategies are needed to explore HIV risk reduction, such as “sero-sorting”, which means choosing a sexual partner with the same HIV status [22,23].

Another important finding of this study was the relationship between years diagnosed with HIV and active syphilis infection. Other studies reported sexual behavior decreased following HIV diagnosis among PLWH; this was also reported in the surveillance system in our province [24,25]. In this study, years diagnosed with HIV was significantly associated with an increased risk of active syphilis infection. Compared to PLWH for a longer time, patients recently diagnosed as HIV-positive are more likely to have intense psychological struggles, and experience emotions of denying, ignoring, and accepting [26,27]. Sexual behavior and risky sexual behavior are reported to initially decrease after HIV diagnosis, but then increase again with increased self-acceptance [28]. The results also revealed that risky sexual behavior persisted after participants were diagnosed as HIV-positive. This is important for public health because transmission will continue among MSM.

We also found that those who were older and diagnosed with HIV infection for a longer time were more likely to be receiving HAART. Consequently, more attention should be focused on those patients to reduce syphilis and HIV transmission.

There are limitations to this study. First, this was a cross-sectional study, making it difficult to determine the relationship between related factors and active syphilis infection. Second, not all participants had syphilis testing until December 31, 2015, which may lead to selection bias. However, the large sample size in this study makes selection bias less likely. The large sample size also helps to identify the possible significant factors. Longitudinal studies are needed to further explore the causal relationships.

## 5. Conclusions

Active positive syphilis infection was high among HIV-positive MSM in Zhejiang Province, indicating a high rate of risky sexual behavior. Age, number of sex partners, years diagnosed with HIV, and HAART were identified as risk factors. Patients who are elderly, live with HIV for a longer period, have more sex partners, and receive HAART should be a focus for interventions to promote changes in behavior and decrease syphilis infection.

## Figures and Tables

**Table 1:** Sociodemographic characteristics, risky behavior, disease progression, years with diagnosed HIV, CD4+ T cell count, HAART, and relationship with active syphilis infection among MSM living with HIV.

Variable	Total n = 3616	Active Syphilis Infection		χ^2^	p
		n = 407	Percentage (%)		
Age (years)				18.214	<0.001
18–25	558	34	6.1		
26–35	1545	193	12.5		
36–45	887	102	11.5		
>45	626	78	12.5		
Marital status				0.678	0.410
Married	830	100	12.0		
Unmarried, divorced, or widowed	2786	307	11.0		
Education				0.960	0.327
Junior high school or less	1422	151	10.6		
Senior high school or more	2193	256	11.7		
Mobility ^A^				0.676	0.713
Between provinces	1327	142	10.7		
Among provinces	406	46	11.3		
Local resident	1883	219	11.6		
Area of registry				0.646	0.422
Zhejiang Province	2289	265	11.6		
Other province	1327	142	10.7		
Condom use with every intercourse in the past 6 months				2.537	0.111
No	691	64	9.3		
Yes	2925	343	11.7		
Number of sex partners in the last 3 months ^B^				28.459	<0.001
≤5	2091	195	9.3		
6–10	839	109	13.0		
>10	523	90	17.2		
Disease progression				4.383	0.036
HIV	2233	232	10.4		
AIDS	1383	175	12.7		
Years with diagnosed HIV (years)				14.259	0.001
<2	1738	163	9.4		
2–5	1469	182	12.4		
>5	409	62	15.2		
CD4+ T cell count (cells/mm^3^) in the last 3 months ^C^				1.364	0.714
≤200	394	47	11.9		
201–350	980	118	12.0		
351–500	1201	133	11.1		
>500	1035	109	10.5		
HAART				12.349	<0.001
No	519	35	6.7		
Yes	3097	372	12.0		

^A^ Mobility refers to inconsistencies between the area registered and residence location; ^B^ 165 missing values; ^C^ Six missing values; HIV: human immunodeficiency virus; MSM: men who have sex with men; AIDS: acquired immune deficiency syndrome; HAART: highly active antiretroviral therapy.

**Table 2:** Multivariate logistic regression analyses of the risk factors associated with active syphilis infection.

Variable	B	SE	AOR (95% CI)	p-Value
Age (years)				
18–25			1	
26–35	0.667	0.237	1.948 (1.224–3.100)	0.005
36–45	0.596	0.251	1.815 (1.109–2.970)	0.018
>45	0.633	0.265	1.884 (1.120–3.168)	0.017
Years diagnosed with HIV (years)				
<2			1	
2–5	0.204	0.136	1.227 (0.940–1.601)	0.133
>5	0.534	0.194	1.707 (1.167–2.495)	0.006
Number of sex partners in the last 3 months				
≤5			1	
6–10	0.506	0.147	1.658 (1.243–2.212)	0.001
>10	0.898	0.157	2.455 (1.805–3.341)	0.000
HAART	0.458	0.221	1.581 (1.025–2.441)	0.039

HAART: highly active antiretroviral therapy; HIV: human immunodeficiency virus; SE: Standard Error; AOR: Adjusted odds ratio; CI: Confidence interval.

## Data Availability

The data cannot be shared because of the confidentiality of the HIV/AIDS patients.
